# Sex/Gender Differences and Autism: Setting the Scene for Future Research

**DOI:** 10.1016/j.jaac.2014.10.003

**Published:** 2015-01

**Authors:** Meng-Chuan Lai, Michael V. Lombardo, Bonnie Auyeung, Bhismadev Chakrabarti, Simon Baron-Cohen

**Affiliations:** a National Taiwan University Hospital and College of Medicine, Taipei, Taiwan and the Autism Research Centre, University of Cambridge, Cambridge, UK; b University of Cyprus, Nicosia, Cyprus and the Autism Research Centre, University of Cambridge; c University of Edinburgh and the Autism Research Centre, University of Cambridge; d Centre for Integrative Neuroscience and Neurodynamics, School of Psychology and Clinical Language Sciences, University of Reading, Reading, UK and the Autism Research Centre, University of Cambridge; e Cambridge Lifespan Asperger Syndrome Service (CLASS) Clinic, Cambridgeshire and Peterborough National Health Service Foundation Trust, Cambridge, and the Autism Research Centre, University of Cambridge

**Keywords:** autism, sex, gender, nosology, etiology

## Abstract

**Objective:**

The relationship between sex/gender differences and autism has attracted a variety of research ranging from clinical and neurobiological to etiological, stimulated by the male bias in autism prevalence. Findings are complex and do not always relate to each other in a straightforward manner. Distinct but interlinked questions on the relationship between sex/gender differences and autism remain underaddressed. To better understand the implications from existing research and to help design future studies, we propose a 4-level conceptual framework to clarify the embedded themes.

**Method:**

We searched PubMed for publications before September 2014 using search terms “‘sex OR gender OR females’ AND autism.” A total of 1,906 articles were screened for relevance, along with publications identified via additional literature reviews, resulting in 329 articles that were reviewed.

**Results:**

Level 1, “Nosological and diagnostic challenges,” concerns the question, “How should autism be defined and diagnosed in males and females?” Level 2, “Sex/gender-independent and sex/gender-dependent characteristics,” addresses the question, “What are the similarities and differences between males and females with autism?” Level 3, “General models of etiology: liability and threshold,” asks the question, “How is the liability for developing autism linked to sex/gender?” Level 4, “Specific etiological–developmental mechanisms,” focuses on the question, “What etiological–developmental mechanisms of autism are implicated by sex/gender and/or sexual/gender differentiation?”

**Conclusions:**

Using this conceptual framework, findings can be more clearly summarized, and the implications of the links between findings from different levels can become clearer. Based on this 4-level framework, we suggest future research directions, methodology, and specific topics in sex/gender differences and autism.

The autism spectrum (henceforth “autism”), a constellation of neurodevelopmental conditions with heterogeneous etiologies,^1^ has been reported as more prevalent in males since the initial case series.^2,3^ This reported sex/gender bias in prevalence has had various impacts on both research and clinical practice. (Note: we adopted the definition from the World Health Organization [http://www.who.int/gender/whatisgender/en/] that “sex” refers to “the biological and physiological characteristics that define men and women,” and that “gender” refers to “the socially constructed roles, behaviors, activities, and attributes that a given society considers appropriate for men and women.” Because most human studies of autism focus on children, adolescents, and adults, it is difficult to separate the effect of sex and gender, as gendered socialization begins at birth. For this reason, unless we specifically refer to “sex” or “gender” as defined above, we use the term “sex/gender” to acknowledge the inevitable overlap between them).^4^ How this male bias relates to the etiologies of and liability to develop autism has been widely discussed, both recently^5^ and 3 decades ago.^6-9^ The downside is that the longstanding underrepresentation of females in research and clinical practice may have generated a male-biased understanding of autism.

Recently, an increasing number of studies from different perspectives and methodologies have revisited how sex/gender differences are related to autism. Some have attempted to clarify how males and females with autism are similar or different in behavioral features via meta-analyses,^10^ multi-site large datasets,^11,12^ and by means of a male/female-balanced design.^13,14^ This has been extended to proteomics,^15^ anthropometrics,^16^ brain structure,^17^ and neural/somatic growth patterns,^18-20^ to name a few levels. On the other hand, studies of population genetics^21^ and genomics^22-26^ have revisited the sex/gender-differential liability hypotheses using well-powered datasets and advanced technology. The use of adequately powered datasets and statistical design as well as multi-level approaches offer promising avenues for advancing our understanding.

However, findings from different studies are complex and do not always relate to each other in a straightforward manner. This is because there are several different (but interlinked) questions embedded in the broad theme of the relationships between sex/gender differences and autism. For instance, asking “Do females with autism have different behavioral characteristics from males with autism?” is different from “Why are there more males diagnosed with autism?” or “Why are males more susceptible to developing autism?” These questions may be interlinked but require different methodologies to address them. Although it is often stimulating to discuss findings from 1 question to address others (e.g., from finding a behavioral difference between males and females with autism, “jumping” to implications for sex/gender-differential liability and etiology), it can be conceptually challenging.

Therefore, we propose a conceptual framework that we hope will help clarify distinct research questions and their interrelationships, aid interpretation of findings to date, and design future research. We first briefly revisit epidemiological evidence for the sex/gender bias in prevalence. We then illustrate 4 different but interlinked levels of research themes, review key findings, and discuss how they may be mutually informative. We conclude by suggesting potential research directions, methodology, and specific topics.

## Method

We searched PubMed for all articles published before September 2014 using search terms “‘sex OR gender OR females’ AND autism.” A total of 1,906 articles were screened for relevance, along with publications identified via additional literature reviews, resulting in 329 articles that were extensively reviewed.

## Results

### Why Link Sex/Gender Differences to Autism? Epidemiology Revisited

The most widely reported male–female ratio for autism prevalence is 4–5:1, lower in individuals with intellectual disability and higher at the high-functioning end.^27^ The association with IQ (a higher proportion of females have concurrent intellectual disabilities) has long been taken as having etiological implications, such as a higher liability threshold for females to develop autism.^6,8^ Most autism studies tend to include participants based on this ratio, or opt to include only males; hence our understanding of autism may have been substantially biased toward males. This problem is evident from the male bias in research samples summarized by meta-analyses: ∼8:1 in brain volumetric studies^28^ and ∼15:1 in task-functional magnetic resonance imaging (fMRI) studies.^29^

Recent large-scale (nationwide), population-based epidemiological studies suggest that the ratio in prevalence/incidence may in fact be lower, in the range of 2–5:1 male:female.^30-39^ Some studies have shown that the sex/gender ratio is not associated with intellectual disability,^31,32^ contrary to previous reports. The trend of lower sex/gender ratio and dissociation from intellectual disability may mean that recent studies have been more successful in identifying higher-functioning females, who may have been missed previously, particularly in clinic- or school-based samplings that are susceptible to ascertainment bias.^40^ This trend may also reflect the broadening of the diagnostic concept that enables more high-functioning females to be categorized on the spectrum.

To confirm the biased sex/gender ratio, it is critical to ensure that the estimation is derived from representative general population samples so as to minimize clinical ascertainment bias, and that the diagnostic criteria and assessment tools are not themselves sex/gender biased.^41^ The relatively smaller male bias in recent large-scale studies is therefore important: the samples are from general population or nationwide cohorts, and some use screening instruments that may be better at capturing subtle presentations in higher-functioning individuals.^42^ It is therefore likely that the male bias, although it exists, is less pronounced than was previously believed.

In brief, the 4–5:1 male bias may be partly due to the underrecognition of females (particularly higher-functioning), ascertainment bias, and issues of diagnostic instruments. Nevertheless, even studies that better account for these issues still show a 2–5:1 male predominance, which has important etiological and developmental implications.

### Four Levels of Research Themes: Setting the Scene

**Figure 1:**
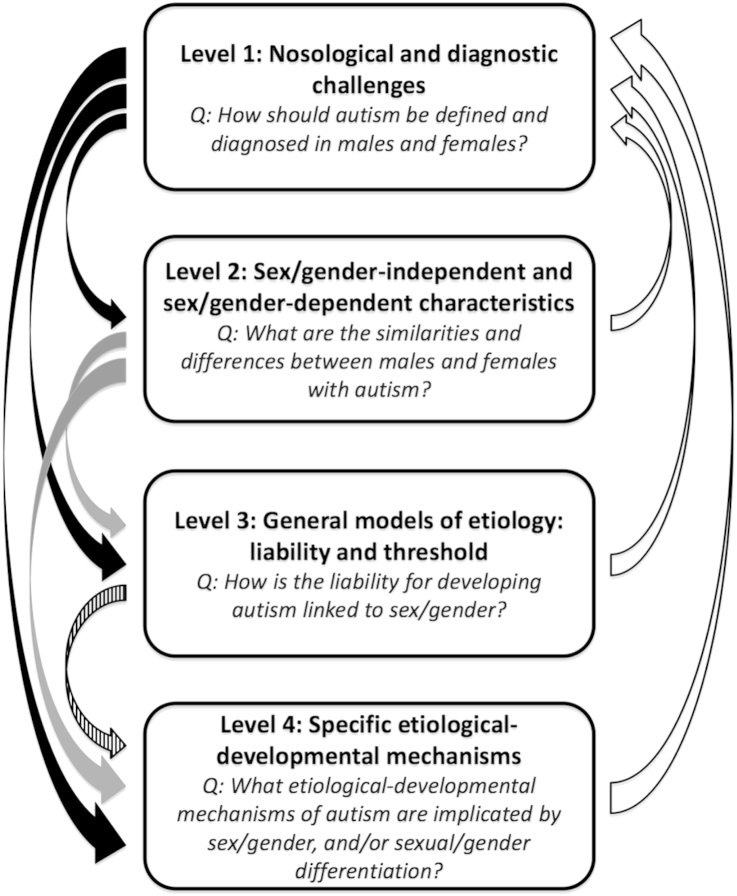
The 4-level framework. Note: This conceptual framework comprises 4 levels of research themes (in bold) and main research questions (in italics). They are distinct but interlinked and mutually informative. Level 1 affects the discovery and interpretation of findings at all other levels (black arrows). Level 2 findings can contribute to the formulation, testing, and revision of etiological models and mechanisms (gray arrows). General etiological models from level 3 can enlighten investigation into specific mechanisms at level 4 (striped arrow). Finally, all findings from levels 2 to 4 can feed back to level 1 reflection (white arrows) for the process of epistemic iteration.^64^

We propose a conceptual framework to clarify the multiple themes underlying studies of the relationships between sex/gender differences and autism. This includes 4 levels that, although distinct, are interlinked and mutually informative (Figure 1).

### Level 1: Nosological and Diagnostic Challenges

This fundamental level concerns 2 challenges in studying any issue involving both males and females with autism: how autism is defined (the nosological challenge) and how autism is identified (the diagnostic challenge). This is a theme that receives the least research attention to date.

### Nosological Challenge

As with most psychiatric conditions, autism is a behaviorally defined syndrome. If our aim is to understand the nonbehavioral aspects (e.g., neurobiology, genetics) of autism in both males and females, we face the critical issue of whether the behavioral definition of autism is appropriate for both. All findings are inevitably interpreted in the light of this initial definition. In addition, if we aim to clarify behavioral differences between males and females with autism, we face a circularity issue, since the behavioral criteria defining autism may be already influenced by sex/gender.

The question of whether slightly different behavioral criteria for autism for males and females are needed is challenging. However, careful reflection helps to resolve whether the diagnostic criteria for autism are male biased, and how the field can move forward with greater consensus on what defines autism. It is important to distinguish among 3 levels of measurement when addressing this issue. “Broad constructs” refer to what defines autism a priori at the most abstract level, irrespective of sex/gender (e.g., the *DSM-5* dyad of “persistent deficits in social communication and social interaction” and “restricted, repetitive patterns of behavior, interests, or activities [RRBI]”). The question of whether sex/gender-dependent definitions of autism are needed should be at the levels of “narrow constructs” and “behavioral exemplars.” Narrow constructs refer to fine-grained subdomains, such as the *DSM-5* symptom subdomains (e.g., social–emotional reciprocity), other psychological constructs (e.g., social motivation, attention to detail), or co-occurring issues (e.g., attention problems, social anxiety). Each narrow construct is composed of a wide range of behavioral exemplars (e.g., eye gaze pattern, type of restricted interest, anxiety symptoms).

Three lines of observations are relevant: (1) qualitative differences between males and females with autism^40,43,44^; (2) quantitative differences in the normative distribution of autistic traits between males and females^45,46^; and (3) developmental differences between males and females with autism.^47,48^ By considering these, we may reach a better consensus on whether and how (at what aspects and life stages) males and females may require partly different criteria in defining “having” autism.

#### (1) Qualitative Differences

Anecdotal clinical/autobiographical reports suggest that there may be “female phenotypes” of autism (Table 1).^43,44,49^ Meta-analysis shows that females on average have less RRBI (e.g., on the Autism Diagnostic Interview–Revised [ADI-R] and/or the Autism Diagnostic Observation Schedule [ADOS]).^10^ However, behaviors captured by these “gold standard” instruments might already be male biased because the formation of items contributing to scoring are likely to have been affected by the longstanding male predominance in case identification.^41,48^ The observed differences may simply reflect that the tools are not sensitive enough to capture how females present their characteristics.

Qualitative differences are best examined by how clinically diagnosed males and females differ on narrow constructs and a wide range of behavioral exemplars, rather than only comparing “algorithm scores” on the ADI-R/ADOS. Empirical studies show that females “meet the criteria” in different ways from males. They exhibit better expressive behaviors (reciprocal conversation, sharing interests, integrating verbal/nonverbal behavior, imagination, adjusting behavior by situation) despite similar social understanding difficulties as males,^40^ different manifestations of friendship problems (better initiation but problematic maintenance, overlooked rather than rejected by peers, better self-perceived and parent-reported friendship),^40,50,51^ different types of restricted interests and less repetitive use of objects.^40^ Studies have also suggested additional features that may be more associated with females, such as demand avoidance.^44,52^

By building new instruments that reflect narrow constructs and collect sufficiently broad behavioral exemplars (beyond classical “autistic symptoms” but also associated and co-occurring features), qualitative differences can be clarified psychometrically, to inform autism nosology in relation to sex/gender. For instance, multi-group confirmatory factor analysis or item response theory models can test whether sex/gender differences persist at the narrow construct level, and if so, whether this is due to the lack of female-specific or sex/gender-independent behavioral exemplars that measure these narrow constructs. If narrow constructs are free of sex/gender differences, the need to develop sex/gender-dependent criteria will be obviated; if sex/gender differences persist in narrow constructs, it implies the need for sex/gender-dependent criteria. Collecting a wide range of behaviors beyond existing instruments (that may have been male biased) can also help delineate “core” versus “non-core/associated” behavioral exemplars, or narrow constructs, of autism for males and females, respectively. By examining endorsement rates, lower rates suggest that a behavior or narrow construct is non-core but rather more frequently co-occurring. Otherwise, by building measurement models with broad and narrow constructs and by examining the loading of each narrow construct onto the broad construct, lower loadings suggest that the construct is not core.

#### (2) Quantitative Differences

Quantitatively, behavioral–cognitive traits linked to autism (henceforth “autistic traits”) are continuously distributed in the general population, and the clinical diagnosis of autism lies at the extreme.^46,53^ Given that males and females have different normative distribution of autistic traits,^54,55^ if the definition of autism hinges on statistical considerations, then the threshold for the level of autistic traits for an individual to be considered as “having” autism (although this is not a sufficient criterion) should be sex/gender normed.^45,46^ This has been done in some studies^21^ and is common practice in other fields of medicine (e.g., defining failure-to-thrive by sex-specific growth curves, or anemia by sex-specific norms of hemoglobin). Nevertheless, since autistic traits have mostly been measured by self or other reports to date, rater bias should be taken into consideration before universal sex/gender norming. Developing additional objective measures of autistic traits will be helpful. Finally, sex/gender norming will statistically equalize the prevalence of above-threshold autistic traits in males and females. It is important to keep in mind that, for clinical practice, diagnoses cannot rely solely on statistical thresholds. Concurrent functional impairment, suffering, and the need for services are also necessary for a clinical diagnosis to be made.

#### (3) Developmental Differences

Culture-based gender role expectations may drive girls with autism to adopt more intrapersonal processes to modify their behaviors (e.g., censuring of behaviors, mimicry of salient gender-normative behaviors, emulating social behaviors, adopting social scripts),^48^ for which the peer group or media may serve as a resource for modeling and camouflaging.^49,56^ Sex-linked biological mechanisms also exert developmental effects. Together these may lead to sex/gender-differential developmental trajectories, which complicate how autism is defined at different stages of life for males and females. It is not known to what extent these plausible mechanisms modify cognition and behavior developmentally because of the lack of longitudinal studies of lifespan development in higher-functioning, later-identified females. How sex-differential biological mechanisms and nature–nurture interplay affect sex/gender-differential development is important but remains underinvestigated.

### Diagnostic Challenge

Age of diagnosis is, on average, later in females than males.^57-59^ Given similar levels of autistic features, males are more easily diagnosed with autism than females,^60^ who require more concurrent behavioral/cognitive problems to receive a clinical diagnosis of autism.^61^ These may be related to the nosological issues above. Equally, the phenomena may involve separate issues about identification, reflecting gender-based interpretation bias from sources of referral (e.g., the family, school, or general practitioner) or diagnostician,^48^ such as interpreting social difficulties as “just being shy” (which may be stereotyped as female-typical).^62^ The phenomena may also be due to greater diagnostic overshadowing or substitution in females, by co-occurring/secondary conditions (e.g., other neurodevelopmental disorders, anxiety, depression) or misdiagnoses (e.g., borderline personality disorder).^56,63^ Finally, they may imply different subgroups in females: those individuals with a more “classical” (male-typical) presentation and/or cognitive delay may be readily diagnosed at an early age, but those who are higher-functioning and have atypical, compensated, or masked characteristics might be under- or misrecognized until later in adolescence or adulthood.

Another view is that it is not females who are prone to be clinically late-diagnosed or underrecognized, but rather their need for a clinical diagnosis is less than males, or that the need arises at a later developmental stage (e.g., in adolescence) compared to males. Although it is important not to delay identification of females in need of support, we should also be careful not to pathologize those who are managing and do not meet the functional impairment criteria for a clinical diagnosis, even if they have high-level autistic traits.

### Implications at Level 1

How autism is defined and identified (as a clinical diagnosis and/or as a research construct) substantially affects all aspects of our understanding of autism. The formation of diagnostic criteria and participant sampling biases are interactive in effect.^41,48^ Better understanding of both males and females is therefore critical for male-predominant conditions such as autism. Developing new ways that objectively sample a wide range of behaviors in both males and females (across different life stages) in association with the use of measurement models will clarify whether sex/gender differences in autism exist at the narrow construct or behavioral exemplar levels, and delineate core versus non-core features by sex/gender. Reaching consensus in defining and identifying diagnostic features that are dependent or independent of sex/gender will ensure continuity of research across multiple levels. Nonbehavioral findings (e.g., neurobiology and etiologies) should be interpreted in light of how autism is empirically defined in the first place. Efforts toward sex/gender-balanced understanding, along with constant nosological reflection, are fundamental to the improvement of the psychiatric classification system through “epistemic iteration.”^64^

### Level 2: Sex/Gender-Independent and Sex/Gender-Dependent Characteristics

This level of research aims to delineate the similarities and differences between males and females with autism. One obvious empirical approach is to compare males and females with autism. The underlying rationale is that sex/gender-independent features (and mechanisms, if etiological factors are tested) may reflect factors central to the emergence of autism, whereas sex/gender-dependent features may reflect sex/gender-specific susceptibility and protective mechanisms. Across multiple levels from cognition to neurobiology to epigenetics and genomics, the convergence and divergence of commonalities and differences will inform general and specific etiological models (levels 3 and 4).

### Methodological Concerns

There are 2 methodological issues critical for the interpretation of findings and for designing future studies. First, given normative sex/gender differences in the general population across multiple levels,^65^ directly comparing males and females with autism will be clouded by potential normative sex/gender differences. Therefore, it is important to compare how males and females with autism differ respectively from neurotypical males and females (e.g., using a 2-factorial design). The null hypothesis is that diagnostic effects are not dependent on an individual being male or female, and contrary findings indicate that there are evident sex/gender differences in how autism manifests. It is also important to attain comparable group size of males and females to improve statistical power, which was often difficult earlier when females with autism were less well recognized.

Second, such male–female comparisons can be done in 2 ways: on a single variable at a time, measuring differences in magnitude; and/or on multiple variables taken together, measuring differences in the pattern of magnitude differences (including and beyond magnitude differences). This clarification is important for informing liability models. It has been proposed that females are “more severe” compared to males with autism^66^; contrary to that, we have argued that for the high-functioning end, at least, females are “different” rather than more severe.^13,17^ If the former is true, we should observe that females have the same feature(s) affected by autism as males but with greater magnitude of change, and/or have atypicalities over and above such “male features” when examining multiple variables. If the latter is true, the test relies on examining multiple variables, where females should have different sets of atypical features compared to those of males.

### Summary of Empirical Findings

#### Behavioral Features

Most studies to date have compared males and females reaching clinical diagnostic criteria of autism based on the *DSM/International Classification of Diseases (ICD)*, alongside confirmation by “gold-standard” instruments, so potential bias originating from these level 1 issues should be kept in mind.

Meta-analysis suggests that females on average show social–communication difficulties comparable to those in males but less RRBI.^10^ Large-scale studies also show less RRBI^12,67^ and even greater social–communication difficulties alongside poorer cognitive and adaptive functioning (as seen in the Simons Simplex Collection [SSC]).^12^ However, in high-functioning adults, given comparable childhood autistic symptoms and current mentalizing ability, females present less evident autistic behavior in interpersonal contexts.^47^ Concurrently, females show greater deviation from same-sex/gender controls than males do in self-reported autism-related traits.^68^ These support anecdotal reports that females may, on average, be more likely to camouflage (i.e., mask or compensate for) their autism,^49^ probably by imitating social acts, following social scripts, and systemizing the social world. The extent to which these sex/gender-differential behavioral patterns (e.g., qualitative/quantitative differences in RRBI, camouflaging) are modulated by co-occurring conditions (e.g., attention-deficit/hyperactivity disorder [ADHD], anxiety) or cognitive/temperamental features (e.g., impulsivity, behavioral inhibition) awaits further investigation.

#### Co-occurring Conditions

Studies directly comparing male and female children with autism report more frequent co-occurring internalizing^14,40,69^ or social symptoms^70^ in females, and more externalizing symptoms in males.^14,40^ In high-functioning adults, however, studies tend to find no sex/gender differences.^47,71,72^ Further clarification of sex/gender-differential co-occurring patterns and their developmental changes will inform autism nosology (e.g., subgrouping) and identification, as well as etiological investigations.

#### Cognition

Apart from the longstanding finding of lower general cognitive and adaptive abilities in females with autism as a group,^12^ few studies have investigated specific cognitive differences. In children with autism (but without control groups), female toddlers achieve better visual reception than boys, yet male toddlers attain better language and motor development.^73^ Girls score higher on the Wechsler Intelligence Scale for Children (WISC) Processing Speed index, Coding, and Symbol Search, but lower on Block Design.^74^ In adolescence, females with autism are poorer in response inhibition than female controls, whereas response inhibition in males is comparable between groups.^75^ Teen females with autism perform similarly to their female siblings on Trail-Making and Block Design, but males are worse than their male siblings on Trail-Making, but better on Block Design.^76^ Males with autism are more impaired in retrieving autobiographical specific memories than neurotypical males, whereas females with autism have no impairments.^77^ Among adults, men and women with autism are equally impaired in mentalizing, basic facial emotion recognition, and inhibitory control, whereas only men with autism are poorer on attention to detail and dexterity involving executive functions than neurotypical males, which is not found for women with autism.^13^ In sum, most sex/gender differences are found in executive functions and visuospatial processing.

#### Growth Trajectories

A trajectory of early brain overgrowth in children between 6 and 24 months of age has been reported in autism^78^ (although some findings may be related to biased population norms of head circumference).^79^ This is particularly evident in the amygdala (beyond global brain size differences).^80,81^ However, early brain overgrowth in autism seems to be sex/gender-dependent. Some studies show sex/gender-differential trajectories and regions of overgrowth in toddlers (e.g., a smaller cerebellum in girls but a larger one in boys with autism compared to controls),^82,83^ and different amygdala volume–symptom correlations.^81^ In addition, early brain overgrowth is observed more in boys with developmental regression than boys without, whereas in girls there is no overgrowth, irrespective of regression.^18^

There is also evidence that physical growth trajectories in children with autism diverge from controls without autism in a sex/gender-specific manner. In case-control study samples, early generalized physical overgrowth was noted in boys but not girls with autism.^20^ In population-based cohorts, boys with autism show similar growth trajectories in head circumference as controls, yet girls with autism show trajectories toward reduced head circumference relative to controls. For body length and weight, boys with autism show overgrowth, but girls with autism have similar length and reduced weight compared to controls.^19^

#### Anatomy, Physiology, and Biology

In clinical samples, females show an increased rate of neurological comorbidities than males with autism.^84^ However, for other neural aspects, the brain structural characteristics associated with autism are different in high-functioning adult males and females.^17,85^ This is also observed in neurophysiology, including body movement variability^86^ and neural activation during cognitive tasks.^87,88^

Using multivariate methodology, serum proteomic and transcriptomic studies also suggest that in high-functioning adults, females are different, rather than more severe, compared to males with autism.^15,89-91^ Anthropometric and neuroimaging studies show that high-functioning adult males and females with autism have different directions of shifts from same-sex/gender controls on the masculine–feminine dimension, that females are masculinized, yet males are feminized.^16,17^

#### Genetics

In light of the well-replicated, critical role of de novo mutations,^92^ which play a more substantial role in simplex than in multiplex autism, it is interesting that paternal age (which is associated with increased risk for de novo mutation in the gametes) correlates with the odds of simplex to multiplex autism in females but not in males.^93^ Corresponding to the predictions from the multi-factorial multi-threshold etiological model (level 3) that females with autism have a greater etiological/genetic load (reflecting a female-protective effect), data from the SSC show a trend toward more gene-rich de novo copy number variations (CNVs) in females than in males with autism,^22^ particularly microduplications,^25^ and in functional hub genes.^26^ This is also true for mutations indexed by single nucleotide variants^23^ and complete gene knock-out (in samples beyond SSC).^24^ Furthermore, females with autism are more likely to have highly penetrant pathogenic CNVs and are overrepresented among individuals carrying exonic deletions overlapping fragile X syndrome protein targets.^94^ An increased rate of mutation in females is also found in other neurodevelopmental disorders.^95^

### Implications at Level 2

Because studies comparing males and females with autism to date use the same diagnostic criteria for case definition, implications are inevitably constrained (see level 1). Overall, females are more likely to have concurrent neurological abnormalities, less RRBI, and poorer intellectual and adaptive functioning than males with autism, although the extent to which this is due to potentially male-biased recognition remains unclear. In samples presenting with these features (e.g., the SSC), females possess a greater load of genetic variants associated with autism, as predicted by the multifactorial multi-threshold model (level 3). How this is related to findings on early growth trajectories in other samples is nevertheless not straightforward, as they show sex/gender-differential trajectories, rather than females being simply more extreme (severe) than males with autism. At the high-functioning end, findings across cognition, neuroanatomy, neurophysiology, anthropometry, and proteomics mostly suggest that females are different, rather than more severe, compared to males with autism. It is unknown whether such patterns are also present at the lower-functioning end.

Given the limited literature to date, it may be too early to draw solid implications for sex/gender-independent and sex/gender-dependent etiological–developmental mechanisms. This may be further complicated by concurrent cognitive and neurological abnormalities. For individuals with these comorbidities, females may be more severely affected, with more etiological factors involved; whether such factors are shared with males remains unclear. At the higher-functioning end, sex/gender-differential mechanisms may play a substantial role, suggesting that males and females may even constitute distinct subgroups (phenocopies).^46^

### Level 3: General Models of Etiology: Liability and Threshold

The male-bias in prevalence, in conjunction with findings that females are often more severe in neurological/intellectual disabilities, has led to investigations into how these may reveal autism etiologies in general, especially regarding the female-protective effect. Owing to the major role of genetics,^96^ most models concern genetic liability and are tested by population genetic studies, although broadened etiological models have also been proposed.^97^ There are at least 4 (non–mutually exclusive) models.

### General Etiological Models and Associated Evidence

#### (1) Multi-factorial Multi-threshold Model

**Figure 2:**
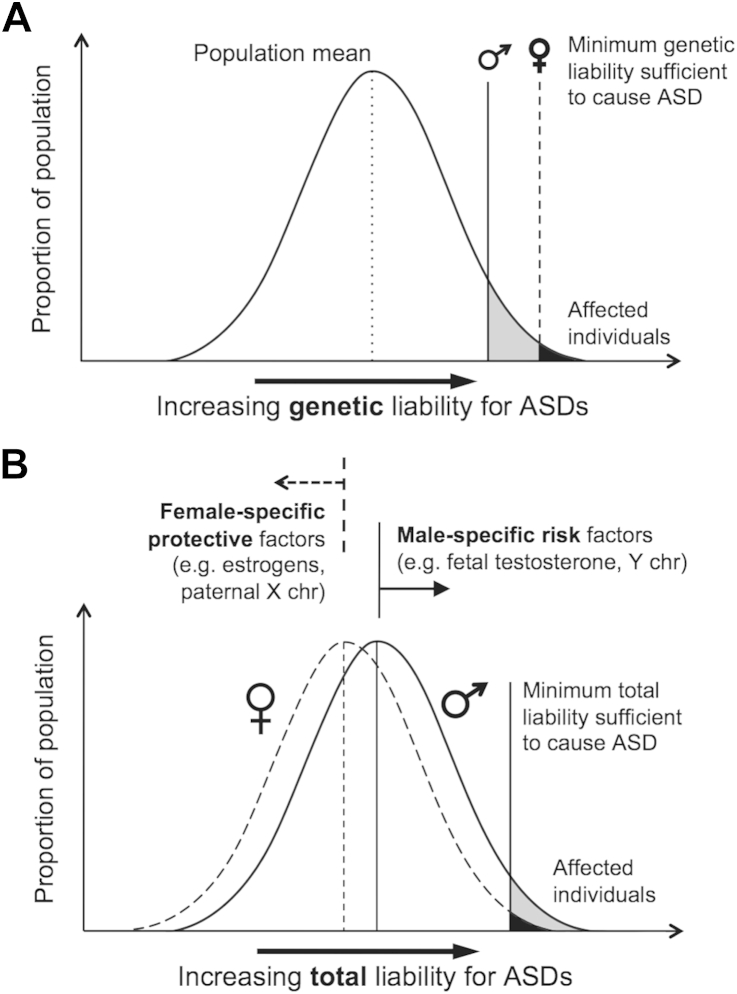
Multi-factorial multi-threshold versus sex/gender-differential liability models. Note: (A) In the original multi-threshold model, genetic liability for autism is normally distributed in the population, and the minimum genetic liability sufficient to cause autism (threshold) is greater in females than in males. (B) In the revised sex/gender-differential liability model, female-specific factors shift females’ total liability distribution (including genetic, environmental, and other factors) away from—and male-specific factors shift males’ distribution toward—a single threshold. ASD = autism spectrum disorder; X chr = X chromosome; Y chr = Y chromosome.

The initial “multi-factorial model of disease transmission”^98^ formulation of autism genetic etiology^66^ suggests that multiple genetic factors (normally distributed in the general population) contribute to the liability for developing autism, and that a higher threshold of such genetic liability is required for females to reach an affected status than males (Figure 2A). This model predicts that there is a higher genetic etiological load in female than male probands, which (assuming familial transmission) should also be carried by relatives, so that relatives of female probands should have a higher load than relatives of male probands. Population genetic studies can test this prediction, assuming that the genetic etiological load is directly reflected in the likelihood of having an autism diagnosis or in the level of autistic traits.

Studies of sibling recurrence rates give inconsistent results. Some support the prediction, finding a higher sibling recurrence rate for female probands,^99^ yet other (larger-sample) studies do not.^100-103^ Additional supportive evidence comes from multiplex autism families, where affected males from families with affected females show more RRBI (but not social–communication symptoms) than affected males from families without affected females.^67^ However, heritability is equal in males and females,^104^ and in familial autism, inherited susceptibility is equally transmitted by unaffected mothers and fathers.^105^

In contrast, studies of autistic traits support the prediction. Replicated across 2 large general population twin samples, siblings of female probands with autism have higher levels of autistic traits than do siblings of male probands.^21^

#### (2) Multi-factorial Sex/Gender-Differential Liability Model

A revision to the multi-factorial model takes into account etiological load beyond genetics.^97^ A key concept here suggests that female-specific protective factors and male-specific risk factors, genetic (e.g., X chromosome gene protective effects)^106^ or environmental (e.g., prenatal hormones),^107,108^ respectively shift the liability distribution for females away from, and for males closer to, the same threshold required for reaching affected status (Figure 2B). This model provides room to test for sex/gender-specific protective and risk mechanisms. It is worth noting that when it comes to etiological implications, theory linking and aligning autism directly with typical sex differences^109^ fits into this model, as it suggests a sex/gender-differential distribution of risks underlying the male predominance.

#### (3) Greater Genetic Variability in Males

A third model suggests that the male predominance and generally less severe symptomatology in males are due to greater genetic variation in males than in females in the general population,^8^ comparable to similar findings regarding other developmental disabilities. This model predicts that more males than females show a high number of autistic features as a result of greater genetic variability, whereas females will develop autism due to additional pathology. Evidence for this latter prediction is inconsistent, with some studies showing increased neurological problems in females^84^ and others not.^66,110^ A direct population-level test is not yet available.

#### (4) Genetic Heterogeneity and Sex-Differential Penetrance

A fourth model focuses on genetic heterogeneity, suggesting that females have a set of genetic etiological factors that are qualitatively different from those in males with autism.^67^ This could be viewed as an example of equifinality.^111^ This model also proposes that the penetrance of autism risk genes may be less in females. Modeling genetic data across 3 large datasets suggests that a simple risk model describing 2 family types (family for whom the risk of autism in male offspring is nearly 50% and family for whom male offspring have a low risk) best fits the data for males; importantly, this model fits the female incidence if a lower penetrance factor of ∼0.3 is added.^112^ The authors propose that sporadic autism is mainly contributed by de novo mutations with higher penetrance in males but lower penetrance in females, and that familial autism is from offspring (often females) who carry new causative mutations but who are not affected themselves.

### Implications at Level 3

Population-based genetic studies are still needed to further test the multi-factorial multi-threshold and the genetic variability models. The multi-factorial sex/gender-differential liability and the genetic heterogeneity models stress the importance of revealing sex/gender-differential etiological structures in autism. For example, modeling sex/gender-differential effects in a genome-wide association study (GWAS) reveals autism risk loci at RyR2 and UPP2 in multiplex families.^113^ Similarly, by GWAS, male-specific autism risk loci have been found at Xp22.33/Yp11.31 (of predominantly paternal origin) in male-only but not female-containing multiplex families.^114^ Across neurodevelopmental disorders, CNV enrichment at 16p13.11 is found for males but not females.^115^ These point to plausible male-specific risk mechanisms. Lower penetrance in females has been shown for a rare CNV (microdeletion) in the autosomal autism risk gene SHANK1 in a 4-generation family, where in males it is associated with high-functioning ASD with or without increased anxiety, but in females it is associated only with increased anxiety.^116^ How sex/gender-differential genetic/etiological structures of autism further account for sex/gender-differential patterns of co-occurring medical, neurodevelopmental, psychiatric conditions and cognitive–behavioral features, and how these conditions or features emerge and evolve, will feed back to level 1 considerations.

How sex/gender-differential etiological structures correspond to cognitive–behavioral and neurobiological findings is also illuminating. Behavior genetics studies of autistic traits show no evidence of qualitative sex differences (i.e., different genetic and environmental influences for males and females), but suggest quantitative sex differences (i.e., the degree to which these influences affecting males and females differ).^117-119^ Phenotypic correlations between the triad of autistic traits (social, communication, RRBI) are higher in males than in females, and this is also true for the genetic overlap among the triad,^117-119^ indicating higher phenotypic as well as genetic coherence in males but more fractionation in females. Furthermore, in the general population autistic traits are often associated with social cognition in males (i.e., higher autistic traits are associated with poorer adaptive coding of face identity,^120^ poorer facial basic emotion recognition,^121,122^ and poorer mentalizing^122^), but not in females. Imaging studies also find sex/gender-differential neural correlates for biological motion processing.^123^ Therefore, an emerging theme is that females may be more resilient to autism because of an underlying more fractionable neurocognitive and genetic architecture. This new general etiological hypothesis awaits empirical examination. In the meantime, we still have to be mindful that all current etiological investigations are dependent on how autism/autistic traits are measured and defined, as reflected in level 1.

### Level 4: Specific Etiological–Developmental Mechanisms

General etiological models help generate hypotheses to pinpoint specific etiological–developmental mechanisms. The conceptualization of female-specific protections and male-specific risks (could be 2 sides of a coin) and sex/gender-differential shifts of liability distribution^97^ implies that examining factors associated with normative sexual/gender differentiation may provide hints about sex/gender-differential liability (and even, but not necessarily, about sex/gender-independent etiologies). The genetic heterogeneity model^67^ suggests the usefulness of clarifying the moderating role of sex/gender in etiology, and the need to examine the extent of common etiologies in males and females. A thorough review of specific mechanisms revealed to date is beyond the scope of this article and is provided elsewhere.^5,48,124^ It is important to note that etiological mechanisms revealed in light of sex/gender-differential liability may not necessarily account for mechanisms shared by both males and females with autism. Sex/gender-independent etiologies need to be tested by demonstrating shared findings in sex/gender-stratified analyses.

### Overview of Candidate Mechanisms

#### Genetic and Epigenetic Mechanisms

An obvious genetic mechanism explaining sex/gender-differential liability are sex chromosomal genes,^5,124^ including male-specific risks by Y-chromosome genes such as SRY (and its downstream effects, including hormonal), and/or female-specific protections from the increased X-chromosome gene dosage in females (from genes that escape inactivation). Associated epigenetic mechanisms related to X-chromosome genes likely further contribute, including skewed X-inactivation, parent-of-origin allelic imprinting,^106^ and hypothetically, heterochromatin sink that results in sex-differential protein-mediated epigenetic effects on autosomes.^124^ Sex chromosome genes (and associated epigenetic effects) may account for only a portion of the etiological mechanisms, as autism risk genes largely involve autosomes.^92^

#### Pre- and Perinatal Environmental Mechanisms

The first candidate is the prenatal hormonal environment (which could be related to downstream genetic effects). Following implications from the extreme-male-brain theory,^5,109^ it has been shown that prenatal testosterone level predicts cognitive–behavioral characteristics related to autism in both typically developing males and females.^107^ Moreover, prenatal steroidogenic activity (hormones in the Δ4 sex steroid pathway and cortisol) is elevated in males later diagnosed with autism^108^; all steroid hormones measured are highly intercorrelated, suggesting that mechanisms that generally alter steroidogenic biosynthesis (e.g., cytochrome P450 enzymes)^125^ are likely atypical rather than specifically just for androgens. In addition, 1 feedback mechanism regulating sex hormonal activity is the RORA gene (and associated molecular mechanisms), which also directly regulates autism risk genes.^126^ These converge to suggest that the prenatal hormonal environment plays a contributing role in etiology. Potential mechanisms include regulating excitatory–inhibitory balance through effects on GABA signaling,^127^ affecting neuro-immune interaction (e.g., microglial activation),^128,129^ or influencing arousal-related amygdala sensitization.^130^ How these effects differ in males and females, however, has yet to be investigated. High-functioning females with autism are more likely to have physiological steroidopathic issues^131^ as well as masculinization of anthropometrics and brain morphology,^16,17^ indirectly implying that hormonal events, if associated with autism, may have greater impact in females.

A second environmental candidate is maternal immune activation, based on its association with the occurrence of autism^132^ and that maternal–fetal autoantibodies are related to subgroups of autism.^133^ How this is sex-differential is unknown, but animal studies show that microglial activation in the developing brain (which may follow maternal immune activation) may be sex-specifically activated by prenatal sex hormones.^128^ This implicates potential joint effects of hormonal and maternal immunologic factors in modulating sex-differential liability for autism.^124^

#### Postnatal Socio-Cultural Mechanisms

Socio-cultural systems in many societies are gendered. An individual’s experience is partly different as a result of gender role expectation and socialization according to one’s birth sex. This may exert gender-differential effects in defining and recognizing autism (level 1).^62^ In addition, gendered experiential effects have also been hypothesized to contribute to sex/gender-differential etiologies by exerting protective effects from increased opportunities for reciprocal social interaction for young girls than boys.^134^ There is as yet little empirical investigation. More studies on how gendered experiences affect the emergence of autistic features in the beginning years of life would be useful (e.g., in high-risk infant studies).

Developmentally, gender may influence how individuals maintain or modify their autism-related characteristics by intrapersonal, family, and social processes.^48^ How a gendered socio-cultural system affects lifespan development (including the development of secondary features) for males and females with autism differentially should be investigated longitudinally.

### Implications at Level 4

Research into specific mechanisms has shown initial evidence in genetics, epigenetics, and the prenatal environment. Genetic and environmental effects are closely entwined through epigenetic and other regulatory mechanisms. Brain gene expression studies show that, although sex-differentially expressed genes do not overlap with autism candidate genes or genes aberrantly expressed in autistic brains, gene ontology enrichment analysis indicates that male-biased transcriptional modules are also implicated by the autism candidate genes.^135^ This suggests that it is downstream pathways that converge to show potential linkage between epigenetic (and genomic) sex differences and autism etiologies rather than individual genes per se. Early prenatal development is a critical period where pronounced sex-differential gene expression and exon use occurs,^136^ and where genetic and epigenetic mechanisms relevant to autism are placing potent permanent neurodevelopmental effects.^108,137-139^ Studies need to go beyond comparing groups at genetic or environmental levels alone, to investigate how their interplay has a role in producing potentially multiple “hits” in the emergence of autism.^124^

## Discussion

We examine the literature linking autism and sex/gender differences and propose a 4-level framework to clarify research themes from nosological/diagnostic issues to etiologies. Given the rapidly increasing interest, we suggest topics of immediate importance to resolve current uncertainties based on this 4-level framework in Table 2.

Although we focus specifically on autism, the principles and issues discussed here could apply to other conditions that show sex/gender differences in prevalence, and/or that potentially have sex/gender-differential characteristics and etiological–developmental mechanisms.^41^ Given the high co-occurrence of neurodevelopmental conditions,^140^ it is important to further examine how the issues raised here apply to other neurodevelopmental conditions and to identify both common and condition-specific issues.

## Figures and Tables

(Reprinted from Werling and Geschwind, Curr Opin Neurol 2013;26:146-153. Copyright 2013 with permission from Lippincott Williams and Wilkins/Wolters Kluwer Health.)

**Table 1:** Anecdotal Descriptions About Behavioral Sex/Gender Differences in Autism^43,44,49^

Domain	Characteristics More Often Present in Females Than in Males
Social interaction	Greater awareness of the need for social interaction
	Desire to interact with others
	Passivity (a “loner”), often perceived as “just being shy”
	Tendency to imitate others (copy, mimic, or mask) in social interactions, which may be exhausting
	Tendency to “camouflage” difficulties by masking and/or developing compensatory strategies
	One or few close friendships
	Tendency to be “mothered” in a peer group in primary school but often bullied in secondary school
Communication	Better linguistic abilities developmentally
	Better imagination (fantasizes and escapes into fiction and pretend play, but is prone to being nonreciprocal, scripted, and overly controlled)
Restricted, repetitive patterns of behavior, interests, or activities	Restricted interests tend to involve people/animals rather than objects/things (e.g., animals, soap operas, celebrities, pop music, fashion, horses, pets, and literature), which may be less recognized as related to autism
Other	Tendency to be perfectionistic, very determined
	Tendency to be controlling (in play with peers)
	High (passive) demand avoidance
	Tendency to have episodes of eating problems

**Table 2:** Potential Future Research Directions

Research Topics	Methodological Considerations
Level 1: Nosological and Diagnostic Challenges	
Nosological reflection on sex/gender-differential criteria: Qualitative	• Qualitative research on female presentations • Developing new instruments that reflect narrow constructs and that collect a sufficiently wide range of behavioral exemplars (beyond classical autistic symptoms but also associated and co-occurring features) • Applying multi-group confirmatory factor analysis (CFA) or item response theory (IRT) models to test for sex/gender differences at the narrow construct level; if existing, testing whether this is due to the lack of female-specific or sex/gender-independent behavioral exemplars that measure these narrow constructs • Delineating core vs. non-core/associated behavioral exemplars, or narrow constructs, for males and females respectively, by examining endorsement rates, or building measurement models with broad and narrow constructs and then examining the loading of each narrow construct onto the broad constructs
Nosological reflection on sex/gender-differential criteria: Quantitative	• Developing objective measures of autistic traits, free from rater bias, to assist the decision about sex/gender-norming • Adopting both sex/gender-independent and sex/gender-dependent statistical thresholds for research related to autistic traits
Nosological reflection on sex/gender-differential criteria: Developmental	• Investigating lifespan development in males and females, especially in relation to sex-linked biological effects and gendered socio-cultural influences
Factors associated with under- and/or misidentification of females with autism	• General population epidemiological studies on autism prevalence/incidence using tools better capturing subtle (higher-functioning) presentations • Epidemiological studies on co-occurrence and shifts of diagnoses over time to elucidate how co-occurring conditions contribute to diagnostic overshadowing or substitution • Exploring how co-occurring conditions or cognitive/temperamental features influence the presentation and identification of autism • Qualitative work to identify mechanisms and consequences of “camouflage” (i.e., masking and/or compensation) • Developing quantitative measures for camouflage • Developing instruments sensitive to females with autism to assist identification

Level 2: Sex/Gender-Independent and Sex/Gender-Dependent Characteristics	
Similarities and differences between males and females with autism	• Using well-powered, comparable male–female group-size study designs • Adopting (at least) 4-group designs to compare how males and females with autism differ respectively from neurotypical (or other groups of) males and females • Longitudinal design to capture differences in developmental trajectories • Multi-level and multi-domain investigation to identify convergence and divergence
Understanding how the findings are influenced by intellectual level and co-occurring conditions	• Comparing above findings in intelligence and co-occurring condition-stratified samples, and including these factors as predictors

Level 3: General Models of Etiology: Liability and Threshold	
Clarifying sex/gender-differential etiological load, threshold, and genetic heterogeneity	• Replicating findings of sex/gender-differential familial (sibling) autism recurrence rate and autistic traits, especially in general-population (rather than clinical) samples • Identifying endophenotypic, genetic, and epigenetic factors associated with the increased diagnoses/traits in families of female compared to male probands (if confirmed) • Examining the above issues in large infant-sibling samples to reveal associated characteristics early in life • Using data from multiplex families and families with broader autism phenotype to compare male-only, sex/gender-mixed (female-containing), and female-only families • Examining how co-occurring medical, neurodevelopmental, psychiatric conditions, and cognitive-behavioral features are related to sex/gender-differential etiological structures
Testing sex/gender-differential shifts of liability distribution	• Investigating what normative sex/gender differences in the general population constitute risk and protection for developing autism (e.g., by identifying sex/gender-differential correlations between autistic traits and biological/neurocognitive characteristics) • Testing whether females have more fractionable underlying cognitive and biological structures that provide better protection

Level 4: Specific Etiological-Developmental Mechanisms	
Whether normative sex differences in genetic, epigenetic, and pre-/perinatal environmental factors contribute to autism etiologies	• Testing and identifying overlap between normative sex differences and known autism characteristics, including downstream effects from both • Investigating multi-level characteristics related to autism in individuals with clinical conditions of altered sexual differentiation • Epidemiological studies to test whether factors associated with sexual differentiation contribute to risk/protection for autism separately for males and females
Whether gendered socio-cultural factors contribute to the emergence, lifespan development, and identification of autism	• Testing whether early gendered environment (e.g., interaction style) affects the onset of autistic features in high-risk infants • Investigating whether gendered environment modulates lifespan development of individuals with autism • Investigating how gendered socio-cultural contexts (e.g., gender stereotypes) influence autism diagnosis in the real world and cross-culturally
Moderating effects of sex/gender in etiologies	• Testing for moderating effects of sex/gender and comparing findings stratified by sex/gender • Identifying sex/gender-specific etiological factors • Investigating whether gene–environment interplay produces multiple “hits” in the emergence of autism, and how sex/gender moderates this
